# Selections and Abstracts

**Published:** 1903-09

**Authors:** 


### SELECTIONS AND ABSTRACTS.

### Criminal Abortion.

The question of race propagation is one that is now attracting the attention of all thinking people of the more civilized nations of Europe. In France grave concern is being felt by its most advanced statesmen for the future of that nation on account of the gradual and continuous diminution of the birth-rate. The recent marked increase of interest among theologians, especially of the Catholic and English churches, of both England and America, on the subject of divorce is an indication of the spread of this thought in those two countries. In our own country, too, the decided attention given to the subject by sociologists, and the emphasis given by President Roosevelt, in his typical term, "race suicide," has no mean significance.

The history of the world is a series of consecutive repetitions. In the early days of the people fertility is characteristic. As the type of civilization becomes more cultured, and as wealth increases, we find this characteristic to decrease. There are, of course, very may things which act as causal factors in this decrease. The obstacles to marriage are multiplied, and family relations become less numerous and are put off until a later period in life. With a diminished religious fervor and the idea that marriage is a civil contract and not a religious sacrament come a diminished opinion of its sacredness and an increased facility for its abrogation. Then, too, any departure from the natural and simpler conditions of animal life brings with it a decrease in the fertility of the female sex. This is true of all animal life and is not restricted to the human race. That this element is making itself noticeable is evident by the articles recently appearing on the subject by members of the medical profession, and has been especially emphasized in the last year or two by the writings of Dr. George J. Englemann of New York.

A not inconsiderable factor in the diminished birth-rate of civilized peoples is the increased prevalence of the practice of abortion. How great this prevalence is only members of the medical profession, and perhaps the priesthood, know. No proper conception of it is obtained by the general public through the occasionally reported deaths which may be directly ascribed to this practice. That it is wide-spread the physician knows, and that it seems to be on the increase is a general opinion among the profession. This forms more than ample justification for its special consideration by the profession, as was done in a Symposium on the subject before the Medical Society of the City and County of Denver at a recent meeting.

As was suggested in the symposium, the prevalence of this practice is due probably partially to the fact that there is a great and wide-spread difference of opinion on the subject of abortion. This is evident in many ways. While there are few who will publicly advocate it as permissible in general, in private life the reverse obtains.

There are many, very many, who, while differing as to what constitutes proper justification, will not only assent to, but maintain, the proposition that, under certain circumstances, it is not only justifiable, but even desirable. Women, particularly married women (and it may be emphasized that abortion is the crime of the married woman and not the unmarried), spread the propaganda among their own sex and tell each other the names of the physicians who have "straightened them out," the method employed, and the fee charged. They will also, very frequently, inform their attending physician, although perhaps not requesting or even wishing such service from him, what members of the profession have, in the past, performed this complacent service for them.

Those who will publicly acknowledge their belief in the justification of this practice are not the only ones who do believe in it. Many, very many, women who publicly, with perhaps great vehemence, condemn it under any circumstances, are not infrequently to be found secretly consulting physicians, under the seal of professional confidence, requesting relief when they find themselves confronted with the evidences of approaching maternity. While the practice is undeniably wrong where others are concerned, it is so easy to find ample and sufficient justification for it in their own particular case—the family is already large enough, they can not afford another child, it will interfere with business and social plans of the greatest importance, the mother's health will not permit, although the physician may not be able to find a single flaw therein, etc., etc., etc., *ad infinitum et ad nauseam.*

Neither religion nor morals are sufficient to deter them, and they regard their own desires as sufficient justification for asking the physician to place himself in danger of trial for murder and a sentence to the penitentiary.

It was suggested by one of the speakers in the symposium that perhaps this difference of opinion extended to the medical profession. This suggestion met with immediate and energetic protest, as was naturally to be expected. Notwithstanding this protest, however, it may safely be concluded that this difference of opinion does exist in the ranks of the profession, although, for obvious reasons there would be greater hesitancy in acknowledging the fact. No physician is willing to be recognized as a professional abortionist, and all physicians recognize the fact that they are liable to be misunderstood unless they publicly, at least, profess the greatest abhorrence for the practice. There is, however, sufficient evidence that such difference of opinion does exist. There is no large city in which there are not members of the medical profession, prominent members at that, and otherwise holding positions of esteem and reputation, of whom it is whispered about that, with proper inducements, relief from an unpleasant condition may be secured, *tuto*, *cito*, and possibly even *jucunde.* It is not altogether unknown that even some of those who are perhaps loudest in their protestations may make special exceptions in favor of certain cases, even where no greater motive than convenience may actuate the patient.

The great prevalence of abortion is due to the trend of modern civilization. Any civilization leading to predominant hypocrisy is, in so far, a corrupt civilization. In sex matters our present civilization falls in this category. It encourages, nay practically compels, hypocrisy in every adult woman not a psychic eunuch.

Any civilization tending contrary to the laws of nature is an infamous one. It should be borne in mind that in nature the sole excuse for the existence of woman, *as woman*, is maternity. Consequently maternity, in and for itself, under whatever circumstances, should be holy, and the badge of approaching motherhood a token of honor and never of shame. Furthermore, in all nature the human animal is the only species (except for the accident of captivity) in which there is failure of even an inconsiderable minority to secure satisfactory mates, and in the human race it is only among the so-called civilized peoples. Among the savages, as among the lower animals, the old maid is a thing practically unknown. Advanced civilization, with its increased social and official demands, renders mating, under the seal of its approval, more and more difficult, and condemns as vice every compliance with the law of nature outside the pale of its hypocritical "holier than thou" brand of pseudo-morality. The respectable murderess will point the finger of scorn to her sisters who, without the seal of the law and with less knowledge of means of escape, is advancing toward the fulfilment of her highest earthly mission. It is justly said that the married woman who interrupts pregnancy is far more contemptible than the single one who, either through ignorance or otherwise, bears the result of her indiscretion. The latter might find some justification for avoiding her natural vocation in the heavy judgment sure to be dealt out to her. The former, in such a vast majority of cases that the exception is insignificant, advances to a crime with apparently little hesitation.

In the text-books, medicine and theology recognize as the only justification for feticide necessity in order to save the mother's life. Practically, this is not carried out in all strictness. Even without feeling that a wrong has been done, it is very frequently the practice to consider the welfare of the fully developed individual superior to that of the immature. Some of the laws recognize a difference in the degree of offense dependent upon the period of gestation, and justification is frequently found for this. Here, however, we are treading upon dangerous ground, Really should any period or definite time be set? If so, what should be the criterion, and why? Why should there be a different penalty, as prescribed by the laws of some States, for abortion after quickening than before? There is certainly no more life within twenty-four hours after that moment than a week or a month earlier. There is scarcely a perceptible difference in vitality, and viability certainly does not date from that instant. The embryo is not one particle more a human being from that time on than it was before.

If it is not possible to fix upon and give a definite time prior to which abortion is allowable then, morally, there is no defense for it at any time. The embryo is a living being from the moment of conception.

It is but a step from the sublime to the ridiculous. To carry the argument out to its logical extreme will draw us from the solid ground of what is practical to the theoretical, and frequently renders the whole argument absurd. This tendency seems inherent in theologians and logicians. It was insisted upon by the reverend gentleman who took part in the discussion before the Medical Society of the city and county of Denver, that *coitus reservatus sue interruptus* falls under the same bann of the church that abortion after conception does. It is a potential abortion, an abortion *in posse.* The ovum is certainly a living being, also the spermatazoon, and therefore as such, entitled to life. But where does this argument carried out to its logical extreme lead us? As was suggested in the discussion, all who do not marry are then equally guilty. To carry it a step further, every youth, who has passed the period of puberty and has had emissions, voluntary or involuntary, is likewise guilty, also every maiden who ovulates without conception. It must be remembered that the God of nature is the greatest sinner in this respect. In every female child at birth there are about 120,000 ova. This gives us nearly 120,000 potential abortions in each female child born. The number of spermatozoa developed in each male during life is untold myriads, and therefore the God of nature provides for untold myriads of potential abortions in every male child born.

To carry the argument further, every believer in evolution must believe in the unity of all life. Not only is each human individual's life related to every other human life, but also to every other form of life, animal or vegetable, and ultimately it is the same in the original, primeval germ of life, amebic or otherwise. Consequently any interference with procreation, animal or vegetable, must be classed together and subject to the same moral laws. How many thousand grains of wheat, for example, are created to one that germinates? How wasteful and how morally delinquent must be the great Creator, if we are to apply our finite logical syllogisms to this subject!

If the Creator cares so little for the perservation of the individual life, and particularly the undeveloped life, why need we, the creature, concern ourselves about it. Here then we have a justification for the whole practice of abortion. Simple, is it not? It is so practical also, and so absurd.—*The Colorado Medical Journal.*

---

### SOME THINGS THE OBSTETRICIAN MAY DO TO ASSIST THE LYING-IN WOMAN, AND SOME THINGS HE HAD BETTER NOT DO.*

By way of explanation, I would say I wrote the following paper just as I would write a letter to a friend. I did not refer to any authors or book, but wrote my thoughts down just as they came to me; thus you will understand why it is I seemingly jumped, as it were, from one subject to another. I did not expect to advance any new ideas, but to call attention to some things that may have passed out of the mind of the busy practitioner.

It is not my intention to attempt to write a paper to teach how to attend a case of obstetrics, but to mention some things that will be of advantage to the patient, as well as the obstetrician. When the obstetrician is called to see a lying-in woman, he should enter the sick-room quietly, speak pleasantly to the patient, ask her how she is feeling. If everything is going on quietly and smoothly, after he has paid his respects to those in attendance, he should call for water that has been boiled and not cooled with cold water; to this he should add his bichloride tablets, and then bathe and wash his hands thoroughly, and if he has not previously pared his finger-nails, he should do so now, and get all of the dirt from under them. If he has whiskers, he should also give them a bath and disinfect them.

*Read by T. J. Shoemaker, M.D., before the Kentucky State Medical Society, April, 1903.

When he has everything aseptic he may walk gently up to the bed and ask the patient to permit him to examine her, that it is very proper that he should make the examination to ascertain her condition, that he will not cause her any extra pain. The examination should be made in the most gentle manner, with the index-finger, first lubricating it with pure fresh olive oil. A well-educated index-finger will make known many things to the accoucheur; whether the os uteri lies low or is high up, dilated or dilatable, whether in a normal position or turned back toward the hollow of the sacrum, and many other things. If pains are severe and the os uteri is turned back, they will never do any good until the accoucheur brings the os uteri down straight and holds it in position. This is not always so easily done. Sometimes you may succeed in reaching the os with the index-finger, and sometimes you can not. Then we might try turning the patient on her left side and by pressing down on the top of the uterine tumor with the left hand, we may be able to reach the os with the index-finger of the right hand. When we once get it in the proper position we should hold it there until the pains have sufficiently dilated the os for labor to go on in a regular manner. Sometimes when the pains are slow and weak we may start them up by titillating the os in a circular manner, or when the os uteri is low enough, one might stretch the os by using the index-finger and its twin brother; this will increase the pains as well as dilate the os. Sometimes we can increase the pains by pressing on the perineum with the index-finger when the pain is on or when we want to bring on the pains.

Sometimes in a lifetime we may run across a "crow-quill os uteri." We will about come to the conclusion that the woman's os has grown entirely up, and that it has been hermetically sealed up, as it were, after the pregnancy has taken place. But do not despair, time and perseverance will help you out, and you will eventually find the little spot high up and far back that you can make some impression on with the end of the index-finger. And when you once succeed in getting the end of the finger into the os uteri never take it out if you can possibly help it until you have brought it down and forward. Do not ever let your finger forget its cunning, but do not be too officious when labor is progressing regularly, although we may hasten labor by titillating the os and thus increasing the pains.

It requires some judgment about giving chloroform in labor when those cutting and grinding pains are dilating the os. Sometimes a little whiff of chloroform will take off the rough places with the nervous lying-in woman, just as a drink of good Bourbon whiskey will make the stones and clods smaller when one is riding over a rough country road in a buggy. But you can not at all times keep up chloroform to an advantage. Sometimes it will stop the pains entirely, and you will be compelled to abandon it absolutely. There is one thing I would warn the young accoucheur against; when you have a primipara case of labor and the pains are going on lovely and the os uteri is not only dilatable but dilated well, and the bag of waters well formed, and you feel like you are going to have a chance once more to be free and sit beside your best girl in the near future, and pour into her ear tales of love, or if you are just married that you will soon be by the side of your darling little wife, do not in your anxiety be tempted to rupture the fetal membranes. If you should become so unguarded at an evil moment you will have, I fear, many hours to regret it. The pains will stop suddenly, everything will come to a standstill, you will think about your past troubles and your sins and dead relatives that were once so dear to you; you will think your past troubles were small compared with how foolishly you have just acted. Your fondest hopes have all departed and blasted, and you are doomed to sit like a condemned criminal until nature may, after a long time, return to action for your relief. The accoucheur had better wait and permit the baby to be born with a veil over its face than to do such a rash thing. Let the bag of waters help the labor as long as it will hold on. Of course, when you have a multipara woman in labor, when the os is well dilated and the bag of waters well formed, you may take advantage of a hard pain and rupture the membranes, and labor will go on lovely. Even if it does not, you can give a drachm of Squibbs' fluid extract ergot and repeat if necessary in a reasonable length of time. But I would not advise more than two doses of ergot, for fear of harm to the child *in utero*. If that does not do the work, desist, and either wait a reasonable length of time or put the patient under the influence of Squibbs' chloroform and deliver her of the child at once with the obstetrical forceps.

I think it is a very good practice if the labor has been tedious to give just before you deliver the placenta a teaspoonful of fluid extract of ergot to the mother and see that the placenta and membranes are entire. Be careful to turn out all the blood-clots from the womb and vagina. I think it is a good plan to keep up a strict watch over the pulse after confinement for an hour after the child is born, and to place the left hand over the womb and keep control of it, if you suspect there is going to be any post-partum flooding. You can always tell then what is going on. You will have less trouble than to let the womb get away from you and have to introduce the hand and arm up about the region of the stomach to bring away blood-clots and induce the womb to contract even by holding a lump of ice in place in the body of the womb. After you have had a case of post-partum flooding and have gotten the womb back to its proper size, next time you would be more than willing to hold it in the hollow of your hand for an hour. One never forgets a bad case of post-partum flooding; that pallor of the face will haunt you for a long time thereafter.

I want to say something to you about support to the perineum, so-called. When you have extra hard pains to contend with, and the head of the child is advancing rapidly toward the perineum, you don't want half-a-dozen old wiseacres standing around yelling to the lying-in woman to shut her mouth and put her chin down on her breast and bear down the pains. The accoucheur wants to be master of the situation himself. Now, it is the head that needs the holding back, to regulate its pressure against the perineum, which the accoucheur can do with the index and its twin finger of the right hand, when the pains are at their hardest, until the perineum is perfectly stretched. If you depend on supporting the perineum from the outside before it is properly stretched, you may have the chagrin of feeling it divide beneath your hand. Of course, after the perineum is properly stretched, it is all right to support from the outside.

I want to say something about the lying-in woman getting tired of and vexed at the accoucheur's meddlesome assistance. Perhaps many of us examine our patients too frequently. But some women are not satisfied if you are not around them, examining them, etc. They think you are not doing anything for them. But there are others that do not want it, and they may say some ugly things to the accoucheur or about him. Then he might quietly withdraw and let her alone if everything is going on all right and he can leave her safely. He may employ his time talking to the other ladies about the room or pick up something and pretend to read, just any old thing will do—an almanac if you can not find anything better. In a short while she will come around all right. You will hear her call for help, and if no one pays any special attention to her you will hear her cry out, "Oh, I am going to die!" Pretty soon if she does not call for you some one else will say "Doctor, do something for the lady, please."

I want to say something to you about the use of chloroform in cases of labor. There are but two objections to its use so far as I know, if the patient can use chloroform at all, viz., it sometimes stops all pains and the obstetrician may have to abandon it altogether, and I think a long-continued use of it in a case of labor may cause post-partum flooding. I think in those cases it is best to give ergot hypodermatically before delivering the placenta, or if the stomach is not sick, a drachm of fluid extract ergot by the mouth. I always use Squibbs' chloroform for inhalation.

I want to say something to you about the delivering of the placenta, and that will close this paper. When I attended medical college they taught that the accoucheur should wait for a time, say for an hour, after the child was born before delivering the placenta. For years I have always delivered the placenta as soon as I could remove the child. If the child is strong it is not necessary to wait until the pulsations stop entirely in the umbilical cord. I tie the cord and remove the child. Keep a strict watch over the uterus, and when the child is out of the way make gentle pressure on the womb and slight traction on the cord, and ask the patient to blow against the palm of the hand. This procedure throws the diaphragm down and assists in bringing on contractions of the uterus. The practice of the old mid-wife of having the patient to blow in a bottle will accomplish the same thing. I have never seen any good reason for waiting to deliver the placenta. On the contrary, if you will follow up the contractions caused by labor you will succeed much better than if you wait and let the contractions die down, and then you are not so liable to have the so-called hour-glass contractions of the womb. Labor pains have the same impression on the uterus that electricity has on the muscles of a frog's leg—the contractions will continue after the cause is removed, and thus you will see the advantage of the quick delivery of the placenta. If I am so unfortunate from any cause to have a "bob-tail" placenta, I give the patient chloroform and introduce my hand and deliver the placenta without delay.—*American Practitioner and News.*

---

### A FEW PRACTICAL THERAPEUTIC FACTS.*

My explanation for reading a paper in which well-known, but plain, facts are stated, and which will have nothing in it but what we all have heard and read over and over, is partly due to the dryness of the subject and varied field which it covers. I do not desire to introduce any new theories or practices in applied therapeutics, but merely by calling your attention to a few practical therapeutic points, freshen your memory along this line, and if this is accomplished its object will not have been in vain.

A practitioner of to-day is beset with so many temptations in the way of proprietary drugs, which are being introduced by the manufacturing pharmacists (which are beautiful in color and pleasant to the taste and dispensed in combinations, in some cases better than we can prescribe ourselves), that the every-day, ordinary drugs and their uses are being gradually shelved more and more.

How many of us stop to consider in the use of the common, every-day drug spirits of nitrous ether, that the results obtained depend largely on the method of administration—*i. e.,* as an antipyretic in febrile affections it should be given in doses of twenty to thirty min. every half-hour. To produce diuresis the drug should be associated with some other diuretic and given in large doses from one to two drachms every three or four hours. If the

* Read by Chas. A. Labenberg, M.D., Richmond, Va., before the Richmond Academy of Medicine and Surgery, June 9, 1903.

drug is desired for its diaphoretic action it should be given in hot water, twenty or thirty min., and the dose repeated every half-hour, patient in the meantime being well covered. As a nervous stimulant the dose should be large, never less than one drachm.

In the use of the bicarbonates to reduce the acidity of the urine the drugs should always be administered after meals. This is a point we all know, when our attention is called to it, but we often prescribe the drug for gonorrhea (where we wish to reduce the acidity of the urine), and the patient is told to take the medicine three times a day, which he may do, and often does, regardless of an empty or full stomach, and if taken after the former will increase, instead of decrease, acidity.

Do we always stop to think that a bitter to be beneficial should be given before meals, and that one bitter should be substituted for another if they are to be continued? If not, the stomach will revolt. Or to think that when the digestion is impaired and the appetite good, it is an indication that the indigestion is intestinal, and therefore, beyond the influence of bitters? Or that in catarrhal conditions of the stomach (as in chronic gastritis or drunkards' catarrh of the stomach) alcoholic preparations of the bitters should not be given, using instead the aqueous preparations, such as infusion, or that the bitters are of no avail in organic diseases of the stomach when the secretion of gastric juice is diminished? When the bitters are indicated we can use the aromatic oils, as their action on the digestive organs is the same as the bitters, and in addition increases the activity of the circulation reflexly by stimulating the sensory of the vagus distributed to the mucous membrane of the stomach.

Don't make the grievous mistake of administering iron in acute inflammatory conditions, or in the anemia of malignant diseases, or in the hemorrhagic diathesis, or to keep up its use when the stomach rebels, or when your patient's rectum plainly indicates by its swollen veins that the drug is contraindicated. It is well to remember that the action of the hypophosphites is the same as phosphorus—only weaker on the osseus and nervous systems and in the treatment of skin diseases—that cod-liver oil is a food, pure and simple, not a medicine, and, therefore, discontinue its use when it proves detrimental to the appetite, causing eructations, heartburn, diarrhea, etc.; that the aqueous extract of suprarenal gland (prepared by using ten grains of the dry extract to two drachms of water and filtered) is invaluable as a pure astringent in all inflammations and as a hematic.

It is well to know that arsenic is the drug par excellence in chorea and pernicious anemia, and especially in the latter disease, as it prevents the destruction of the red blood-corpuscles rather than building up the blood by increasing the number and quality of the red corpuscles; and that next to quinine it is the most potent remedy we have for the treatment of malaria. We should guard against using arsenic in acute skin diseases as much as we would advocate its use in chronic diseases of this character.

In using salicylic acids be careful about a weak heart or weak stomach, as the drug is very depressing. If any benefit is to be derived from salicylic acid in acute rheumatism it must be used early in the disease and in heroic doses at comparatively frequent intervals, not less than twenty grains every two, three or four hours for an adult. If too serious gastric and cerebral symptoms manifest themselves the drug must be decreased in amount or discontinued until the unpleasant action subsides. In any other condition where the drug is indicated it is better to give small doses frequently repeated than to give a full dose at once. While on the subject of the treatment of rheumatism, it is well to remember where there is a weak heart salicin is a much better drug than salicylic acid.

In the choice of your antipyretic, use phenacetin, as it is tasteless, seldom excites nausea, diuresis, diaphoresis or diarrhea, and all in all is about the best drug for reducing fever from any cause. In this connection, I wish to again call your attention to the very important, but often overlooked, point of administering opium to children; that children bear opium badly, as minute doses have been known to prove fatal. As small a dose, as one drop of the tincture, has been known to cause the death of a one-month-old child. If we are compelled to use this drug in a child the best preparation is the camphorated tincture, and largely contrasted to its use is the action of belladonna, children having often taken enormous doses in comparison to adults.

When the use and therapeutics of that important drug ergot are mentioned, it seems as if we allot its field to but one class of diseases, but its uses are manifold and as satisfactory, therapeutically, as nearly any drug in our pharmacopeia. Not only is it a valuable remedy in obstetrical practices and hemorrhages of whatever nature, but of inestimable value in incontinence of urine (in the adult particularly); in cerebral hyperemia and consequent mania, as well as cerebro-spinal meningitis, myelitis and congestive headaches.

In the use of santonin it is important to inform your patient that his urine will be colored yellow, his eyesight may be interfered with and that he may be troubled with a rash. And so it is important to inform your patient in the administration of methylin blue about the effect upon the color of the urine, or you may be aroused early in the morning hours with a frightened patient, as I have been when administering this drug for gonorrhea. And so I might go on indefinitely stating facts, which, as I have said before, are well known to all of us, but are so often overlooked in our daily practice; but my allotment of time is short, and I will simply state, without comment, a few important points: Don't forget that colchicum is the ideal vegetable alterative; that squills should not be used in acute diseases of the kidneys; that expectorants of two classes—sedative and stimulating—one to be used in acute diseases and the other for chronic; that apomorphine in small doses—1-40 of grain—is an exceedingly efficient remedy in acute bronchitis and the hacking cough of bronchitis—but beware of it in children; that in the treatment of all diseases of the respiratory system aconite is the drug par excellence; that the aqueous preparation of digitalis is better as a diuretic and the alcoholic preparations are better as heart stimulants; that strychnin is contraindicated in acute inflammatory conditions of the spinal cord and excites reflex irritability.—*Virginia Medical Semi-Monthly.*

### PARASYPHILIS.

Since the publication in 1894 of Fournier's work in which the term "parasyphilis" was first applied by the author to a group of heterogeneous affections much interest has been aroused. Numerous miscellaneous, atypical lesions, apparently syphilitic and just as apparently not, had long vexed the experience of physicians, and the quasi solution offered by Fournier found ready acceptance.

In his address before the International Congress at Madrid (*Lancet*, June 13, 1903), George Ogilvie dissents from Fournier's views and offers some caustic criticisms. Ogilvie observes that a parasyphilitic affection is defined as one syphilitic in origin but not in nature. Its two characteristic features are the following: (1) Parasyphilitic affections are to be met with independently of syphilis; they may be due to other causes as well, they are banal, while syphilitic lesions, properly so-called, such as mucous patches and the gumma, are never produced outside the domain of syphilis. (2) The so-called specific drugs—i. e., mercury and iodid of potassium—have a totally different influence upon parasyphilitic affections from that which they are known to exercise upon true syphilitic lesions. "There is really an abyss between the one and the other." These drugs are said to have either no effect at all upon parasyphilis or the effect is slow and incomplete, while in the case of syphilis proper it is "rapid and efficient." "These two principal features," according to Fournier, "seem to establish a well-marked line of demarcation between syphilitic affections proper and the so-called parasyphilitic ones."

The diseases covered by this definition are legion; it includes iritis, choroiditis, hemiplegias, neuralgia, neuroses, anemia, giantism, cataract, astigmatism, leucoderma and a host of skin rashes, etc., and even tabes. As Ogilvie says: "One is at once struck by the variety of the pathologic conditions which meet the eye. There are inflammatory processes, progressive degenerations, dystrophic conditions, dyscrasias, scars, tumors, predispositions, anything from a slight anomaly to monstrosity, from imtellectual weakness to complete absence of the brain, from simple uncomplicated debility to "la morte banale," moreover moral defects, functional disorders, diatheses, etc. The method by which this ubiquity of syphilis is established is very simple. Every pathologic condition which is " banal " and not rapidly and intensely benefited by mercury found in a heredosyphilitic subject (or one suspected of heredosyphilis for the reason that one of the parents or grandparents has at one time suffered from syphilis) is registered as parasyphilitic, and subsequently the cases are arranged under different pathologic headings. Of course, in this manner a fairly large number of cases have been collected in which congenital syphilis or a history of acquired syphilis in parents or grandparents has been coexisting with nearly every imaginable pathologic condition. But coexistence is not causal connection, and the question of origin, to be satisfactorily answered, requires much more subtle methods than compilation, enumeration and registration.

Taking into account " the large number of persons of syphilitic ancestry, and the comparative infrequency of transmitted lues, there seems to be little ground for suspecting a deleterious influence of syphilis upon third or fourth generations. This was partially confirmed by Jullien at the Paris Congress.

As to the second characteristic—the influence of specific medication—Ogilvie thinks it merely a question of degree. He says: " Of course in many syphilitic affections the effect of mercury and iodid is at once intense and rapid. But I am not going to enumerate all those in which it is slow, a little or none. Quite apart from cases of idiosyncrasy to the drugs, or cases of malignant syphilis, one has only to think of the many disappointing results in cases of hemiplegia, choroiditis, ophthalmoplegia, spastic spinal paralysis, etc., to realize the incongruity of making the effect of the drug a criterion of the nature of the disease. On the other hand, it is not generally admitted that specific drugs are powerless in leucoderma, tabes and general paralysis. Good results have been reported in general paralysis and in tabes which unfortunately I have never been able to obtain. Fournier himself has had most satisfactory results, amounting to complete cures, in cases of parasyphilis, where one would expect them least—viz., in certain heredosyphilitic dystrophies—and he advocates mercurial treatment in most cases in which parasyphilitic affections are met with in children only suspected of heredosyphilis."

The following quotation from Hutchinson is quite apropos :

"Those who think that the result of treatment by specific may be relied upon as establishing the diagnosis may easily make great mistakes. Sypbilitic affections often resist treatment for a long time (I may add, even altogether) and, on the other hand, mercury and iodid often effect the greatest possible good in cases which are not syphilitic."

Ogilvie's view is summed up in his remark that parasyphilis might be called a pathologic lumber-room; there may be a few valuable pieces in it, but the greater part is worthless.—*St. Louis Medical Review.*

---

### SUMMER DIARRHEA IN INFANTS AND CHILDREN.

In Dr. A. Jacobi's standard work on "Therapeutics of Infancy and Childhood," the author makes the following statement:

"In acute cases of intestinal (or gastro-intestinal) catarrh with high temperature, application of water of from 60 to 70 degrees F. to the abdomen will render good service. The cloth must be wrung out thoroughly, covered with rubber cloth and flannel, and changed when warm. Anemic children and those with much pain require warm or hot applications, which may be preceded by a warm bath. Frequent injections of water of 100 F. or more, with or without an antifermentative, such as thymol (1:1000 or 2000) answer well in most cases.

In great debility or collapse the water ought to be from 105 to 112 F., and contain some alcohol and opium, or teaspoonful of the tincture of musk. The addition of gum arabic to the injection, or the use of glutinous decoctions (flaxseed) instead of water has a satisfactory influence. Starch injections have the advantage of adding to the nutrition of the body by the facility with which the colon changes amylum into dextrin, which will be absorbed. Part of the injected water will always be absorbed, fill the blood-vessels, and may prevent intracranial and other thromboses. Indeed, in many bad cases in which the cerebral symptoms of the so-called hyrencephaloid condition have made their appearance, or are imminent, frequent injections into the rectum of a few ounces of warm fluid contribute considerably to the restoration of circulation."

The above is quoted chiefly to show the high value that is placed upon enteroclysis in the treatment of diarrhea in infants and children by such an eminent authority as Dr. Jacobi. The importance of washing out the lower bowel can not be too strongly impressed upon the general practitioner.

In a communication recently received from E. J. Melville, M.D., of Bakersfield, Vt., he states:

"The season is fast approaching when the wide-awake physician must look up his weapons of defense against the intestinal diseases of childhood. Shall we give digestives when the mucous membrane of the stomach and bowels is inflamed and incapable of retaining any nourishment to digest; or shall astringents be exhibited when to lock up the secretions would be but to add fuel to the flame? Should we risk the danger of opium poisoning in order to temporarily relieve some of the most distressing symptoms or to allay the anxiety of anxious parents? No doubt cases occur when some one or all of the above mentioned remedies are imperatively indicated, but the majority of the patients will recover if strict attention is given to diet and hygiene, and a mild antiseptic used to sterilize the *prima via.* For the past two years I have followed a plan of treatment in these cases which has proved very satisfactory. After due care has been given to cleanliness, fresh air, sunlight and a suitable diet or lack of diet, Glyco-Thymoline is given by mouth and rectum. This preparation has been chosen for the following reasons:

1. It is pleasant to take and thus easily administered to children. 2. Although a mild antiseptic, it has shown no poisonous effect, even when a large quantity has been absorbed. 3. It is the best of good tonics and favors osmosis from diseased surfaces, thus lessening inflammation and promoting healthy granulation in cases where an ulcerative process has begun. 4. On account of the oily consistency of Glyco-Thymoline, it remains in contact with the mucous membrane for a considerable length of time, thus acting in double capacity of a protective and an absorbent. This latter quality is easily explained by the strong affinity of Glyco-Thymoline for the products of inflammation. The following cases may be of interest to the profession:

CASE 1.—Was called to see Mary P., aged eight, on July 4, 1900. Family history tubercular. Pulse, 102; temperature, 100 F. Diarrhea, vomiting, pain, tenderness and tumefaction over small intestines. Dilatation of pupils, loss of appetite, flesh and strength. Night sweats. Other organs healthy. History of recurring attacks every two months for past three years. Gave Glyco-Thymoline, one drachm to four ounces of water every four hours, and high rectal injections in knee-chest position of one ounce of Glyco-Thymoline in a quart of warm water every eight hours, having the patient retain as much as possible. Diarrhea and vomiting controlled in thirty-six hours. Convalescence uneventful. Continued Glyco-Thymoline in thirty-minim doses three times a day for three weeks, when further medication was considered unnecessary. Prescribed an easily assimilated diet, rest in the open air, and cool sponging of abdomen daily. No return of symptoms to date, May 28, 1902.

CASE 2.—Saw G. H. F., aged three months, on August 3, 1901. Cholera infantum. Pulse, 170; temperature, 105; respiration, 44. Vomiting and purging of blood and mucus. Tenesmus of rectum. Symptoms of collapse. Ordered hot saline baths, followed by a brisk alcohol rub every two hours. Discontinued all food for thirty-six hours. Gave hypodermics of brandy, thirty minims, every three hours, and twenty minims of Glyco-Thymoline in one drachm of water at the same time. High rectal enemas of one ounce of Glyco-Thymoline to a pint of hot water three times daily. Vomiting controlled in forty-eight hours and diarrhea much lessened. Gave twenty minims of Glyco-Thymoline in four ounces of broth every four hours, which was retained, and continued rectal injections for five days, when all untoward symptoms had disappeared. Uneventful recovery.

CASE 3.—Was hurriedly summoned on September 8, 1900, to Maggie G., aged four years, who was having convulsions. Temperature per rectum, 107 F.; pulse, 135; respiration, 49. Purging of greenish colored fluid. Stools numbered thirty in past twenty-four hours. Hot mustard bath, followed by a brisk alcohol rub. Mustard to extremities. Glyco-Thymoline, four ounces to three quarts of water as hot as could be borne by rectal injection, allowing the fluid to flow out alongside of nozzle and injecting it slowly. This was repeated every four hours. On the following day the child's temperature was 104; pulse, 138; respiration, 48. No convulsions in past twenty-four hours. Gave thirty minims of Glyco-Thymolide in two drachms of water by the mouth every three hours. Temperature now began to fall rapidly and was accompanied by a corresponding decline in pulse rate, respiration and number of evacuations. Child began to ask for food and was given hot beef juice, two ounces every two hours. From this time on improvement continued rapidly and in four days the patient was convalescent. Continued Glyco-Thymoline for four weeks in twenty-minim doses four times a day, well diluted with cold water. At the end of that period a normal condition was established.

---

### THE INTESTINAL ADMINISTRATION OF MEDICINES.

Gastric interference is yet the stumbling-block in our present system of intestinal medication. We realize this, and, as physiologists, decry the reprehensible practice in vogue of frequently neglecting it. A most excellent treatise has just appeared on the subject and deserves ample review: "L'Administration Intestinale des Medicaments," by Dr. Samuel Bernheim, Paris, France, is an arraignment of our present system and offers a means of overcoming the universal error of committing our drugs to the stomach instead of the intestine, where absorption is alone possible and where needless and harmful complications can be avoided.

The stomach, writes Bernheim, is not an organ of absorption, nor even of digestion, except in an auxiliary manner to the intestine. Besides this, we do not give medicines for the latter purpose and, therefore, such action may be wholly disregarded. The stomach, then, is to be looked upon as a temporary depot in the evolution of an exhibited drug and an agent wholly passive, which can be made no use of save in exceptional cases, in which certain salts in minute quantities are actually taken up.

The problem then is to place our medicines in the intestine unchanged, where they can be absorbed, where they will not be acted upon by an acid and modified into undesired substances.

Many arguments lend weight to these considerations. In the first place, as has been mentioned, it is wrong to place a drug in contact with the gastric mucosa for any length of time. Our preparations are practically all topical irritants and much harm can result therefrom; especial instances of harmful products are many of those met with in current work, *e. g.*, arsenic, ichthyol, the aromatic oils, the salicylates, creosote, iodid of potassium, bichlorid of mercury, phosphorus, the cacodylic salts, etc., from the exhibition of which we so commonly observe anorexia, pain, foul eructations and emesis. This demonstrates the validity of the first premise.

Secondly, a great many drugs are given for a more or less local therapeutic action in the intestines, *e. g.*, purgatives, anthelmintics, certain astringents, digestants, etc., and it is quite needless to elaborate reasons why a sojourn for an hour or two of such a drug free in the stomach would be against all sense.

In the case of purgatives, especially, where the occurrence of nausea and vomiting evidences the fact that the physiologic "enterite medicamenteuse" of Amozan, becomes likewise a "gastrite medicamenteuse," and it will be conceded, very unnecessarily. For, although a degree of congestion of the intestine attends the brisk action of a purge, there is no necessity of inflicting such congestion on the stomach.

Thirdly, there must be considered the inimical effects of gastric juice on medicines, *e.g.*, the conversion of salts into chlorids, the destructive action on artificial digestants, etc.

Fourthly, the harmful effect of most medicines on the peptonisation of foods, the serious digestive disturbances that result because of a modification of the gastric juice formula, from which either the HCL is taken up, or combinations are made with the chlorids of $NH_4$–K—Na and Ca, or phosphates of these bases.

The above considerations are sufficient to the end of arousing an effort to improve on our present system and the attempt has been often made. The most prominent of these will be briefly noted:

Guilliermond, in 1853, introduced dough-cachets for this purpose, but it has been found that they dissolve very rapidly in the stomach and permit the egress of the contained drug before it has passed the pylorus.

Pills covered with various sorts of resinous varnishes were also tried and rejected. Covered with collodion they did a little better, but it was found that fissures in the envelops would occur after a few moments and permit the entrance of the juice and the destruction of the pill in the stomach.

Gelatin envelops or capsules were invented by Mothes, in 1836, and were made of gelatin, gum, sugar, honey, glycerin and water. They present to-day a favorite way of giving many bitter and irritating substances in liquid and solid form. But they present the difficulty of dissolving very rapidly, and in the case of aromatic oils—copaiba, santal oil, etc., do not prevent the most disagreeable stomach complications. Furthermore, the gelatin capsule itself, although soluble, is absolutely indigestable and may be a source of trouble in the stomach. The problem then becomes one of finding an envelop of some sort, insoluble in an acid medium, but readily soluble in the intestine.

Double capsules were tried, but in view of the fact that the time required for the expulsion of ingesta into the intestine from the stomach is so variable the method becomes crude and unfit.

Unna, in 1884, proposed keratin as a protecting envelop, and obtained the substance from the shells of turtles, the upper layers of the skin, the nails and bird feathers. His preparation was free from mucin, fats and mineral matters. Keratin is insoluble in dilute acids and soluble in alkalies—but only in very caustic alkalies and not readily in those found in the intestine. Modifications have been made of it, but always unsuccessfully. Keratin has been practically abandoned.

Envelops of salol have been proposed by Goon, and used; his formula was salol 2.00, tannin 0.50, ether 10.00. This mixture is painted over pills or capsules. But here again fissuring occurs, and there is insufficient protection, or none at all. Furthermore, salol possesses active therapeutic properties and these may at times be entirely undesirable.

It seems, however, that the suggestion of Raquin, which was made some years ago, should be heeded. He proposed gluten, and the work of Bernheim has been to announce the results of his experiments with this substance. It is not possible nor desired to give a complete survey of his extensive work on the subject, other than to say that he has found in gluten a perfectly satisfactory coating, practically insoluble in dilute acids and very readily soluble in dilute alkalies.

The process of preparing the gluten is after that of Fumouze, which consists in having it thoroughly cooked and dried, not fresh nor raw. In this condition it resists the gastric juice for twelve hours. No change at all in the envelop has occurred in five hours' time, while the capsule is entirely dissolved in intestinal juice in the space of from thirty to sixty minutes.

It is apparent then, that we have an ideal substance in which to place the great majority of our medicines, and it is hoped that in the near future such will become common practice, for to-day, a goodly half of the medicines we use are made harmful by our manner of giving them.

There is yet another and, perhaps likewise, important consideration, and this is to preserve the intestinal mucosa from the rapid and injurious effects of medicines, for, although this is the proper point for their absorption and the place at which little harm can result, there is wisely proposed a manner of protecting the mucosa by means of the incorporation of certain absolutely bland resinous substances into the medicine which will serve the purpose of delaying its absorption—of spreading it out more extendedly in less concentrated form. Herein lies a great advantage, which would, however, be practically useless if used without the gluten envelop.

The last half of the book is taken up with clinical and laboratory data which can not be reproduced here, although it will be stated that ample proof is offered from both sources to verify the above claims. Cases are mentioned to illustrate the ease with which the most extreme intolerance of the stomach was overcome, and likewise cases that substantiate the propriety of looking primarily to the intestine for the absorption of our medicines.—*Courier of Medicine.*

### COUGH IN PULMONARY PHTHISIS.

BY J. LEFFINGWELL HATCH, B.SC., M.D., F.R.M.S., LONDON.

As broods silence back of sound, so also stands designer back of design, and the logical mind of man has ever thus traced a presumptive relation between the thing observed and its supposed origin, and called them, respectively, cause and effect.

Thus in medicine we look from symptoms to a cause, and if post mortem we find a definite lesion we too often jump to the conclusion that it must be the very thing we are looking for, and are apt to forget that back of this change of structure lingers the first real cause in perverted physiologic function.

One of the best known and oldest symptoms, and one which occurs from diverse causes, is cough, and this, with another, almost as common and well known, dyspnea, go hand in hand among the various affections of the respiratory organs.

In pulmonary phthisis cough is usually the first symptom manifest and lasts throughout the disease; but the cause is not the same in each stage and consequently requires careful study and varying treatment in the different stages.

The earliest physiologic alteration is a hyperemia usually occurring at the apices. This congestion of the capillaries is the causal irritation that brings about the cough reflexly through the medium of the nervous system. Here a nerve depressant and vaso-motor dilator is indicated rather than an analgesic and expectorant.

In the next stage of consolidation the hepatized tissue acts as a foreign body and likewise reflexly brings about a useless cough in the vain effort to get rid of itself. In this stage resolution should be established by means of an alterative and the nerves quieted by a sedative.

In the third stage where the tissue has undergone cheesy degeneration and broken down, it really is a foreign body that causes the cough, which can only be relieved by its removal, hence we give stimulating expectorants in combination with sedatives and analgesics to relieve the nervous spasms and consequent pain.

The sum total of the forces of a consumptive is at the most a low figure, and we try to keep this up by a high diet that often deranges other organs, whereas regard to the conservation of force by lessening the cough will give the same result without detriment to other emunctories.

To allay cough, then, has been the aim of therapeutists from time immemorial, and of the different concoctions and mixtures that have been vaunted and foisted upon long-suffering humanity their name is legion.

Probably the greatest boon that ever came to us in the form of medicine was opium, and some form or other of this drug has been and always will be used to a great extent as an ingredient in every cough mixture.

Of the alkaloids of opium, morphia has probably been the most popular until recent years, when codein has claimed considerable attention and threatened to usurp its place; but since the discovery of heroin, by Prof. H. Dresser, of Elberfeld, Germany, in 1898, this has been made impossible, and the new analgesic after careful study both in Europe and America has found great favor among practitioners, especially in diseases of the respiratory organs.

In the fall of 1900 my attention was called to Glyco-Heroin (Smith), and I tried a sample bottle on a patient with such gratifying results that I determined to make further observations.

What these results were, the clinical record below tells more graphically than worded phrases of description could hope to do.

It does not nauseate, and can be given in teaspoonful doses as often as every two hours to adults, dose of course being graduated in children according to the age, although they tolerate heroin where opium would produce untoward results.

The greatest advantage this preparation has over all others lies in the fact that it does not contain anything that deranges the stomach, and can be given indefinitely without the patient turning against it.

The majority of cough-mixtures contain sugar, which is bound to undergo more or less fermentation; opium, which constipates and affects respiration, and belladonna, which checks the secretions, so that if they are able to lull the patient into oblivion of his condition for a few hours on account of the large amount of narcotic they contain, he awakes to find a stagnation of secretions with renewed paroxysms of coughing, and "pushing the mustard to fanaticism" for further relief he eventually becomes a slave to opium.

I have used Glyco-Heroin (Smith) now in over fifty cases, with the unvarying result that it relieved the cough, reduced the temperature, increased the volume of respiration, and allayed the night sweats, while at the same time it did not derange the stomach or cause constipation, did not produce vertigo nor nausea, never weakened the respirations, nor caused deleterious effects upon the heart, so that I can frankly say that without doubt we have in this compound the ideal cough mixture for the cough of phthisis pulmonalis.

The cases that I here quote I have selected from a series of fifty-three, with the idea of not citing cases so near alike as to produce monotonous repetition, no matter how gratifying the results.

As has been well said of this preparation, it is not only a true pharmaceutical product but an ethical one as well, and one that the physician can use understandingly, as its composition and physiologic action are well known.

Unfortunately all good things are sooner or later imitated, and something put forward as just as good but cheaper, and Glyco-Heroin (Smith) is no exception to this rule, so if results are not satisfactory, substitution must be at the bottom of it.

OBSERVATION ONE.

Mrs. Marie B., aged 32, father living in good health, mother died several years ago, does not know cause of death.

She was thin, and her complexion was of a muddy, yellow color when first examined. Weight, 122½ pounds; pulse, 100; temperature, 100° F. Respirations, 36 and difficult.

She had a fairly good appetite, but was constipated. She menstruates regularly, but has coughed and expectorated for two or three years. Sputum analyzed showed the presence of tubercle bacilli. She had a pleurisy eight years ago the result of a cold, both lungs were affected since then, crepitant râles throughout, and areas of congestion here and there.

Her sputum had been tinged with blood but she had never had any hemorrhages.

I gave her an emulsion of cod liver oil, and Glyco-Heroin (Smith) in teaspoonful doses every two hours. The cough was relieved from the first and after four months had entirely disappeared. The lungs cleared up, no more râles or areas of congestion, and she gained ten pounds in weight.

### OBSERVATION TWO.

Miss E. M., age 32, unmarried. Had been ill six months before coming to me for treatment, and a diagnoses of tubercular laryngitis had been already established by some one else.

There was dullness on percussion over nearly the entire area of both upper lobes of the lungs, she had night sweats, fever and a persistent cough, raising considerable. She was pale and emaciated, highly excitable and nervous; pulse, 110; temperature, 102° F.; respirations, 26.

Microscopic examination of the sputum revealed the presence of the tubercle bacilli.

On laryngoscopy I found an extensive ulcerative process on the posterior wall of the larynx just above the vocal cords, and both epiglottidian folds were congested and swollen.

Besides the local treatment for her throat trouble and constitution care I gave her Glyco-Heroin (Smith), one teaspoonful to be taken every two hours.

There was marked improvement after the first-twenty-four hours, and she said she had slept well through the night, had coughed scarcely at all, freedom from which distressing symptom she had not enjoyed for months.

The temperature gradually went down to normal, the night sweats ceased, and in a little over one month's time the cough had left her entirely. The ulcer in the larynx was finally healed, which relieved her hitherto painful deglutition; besides this she gained flesh and strength, due, undoubtedly, to the conservation of force which the mitigation of the cough afforded.

### OBSERVATION THREE.

Mrs. I. T., aged 35, had one sister who was tuberculous. She had been ill for over ten years when she came to me; previous to her bad feelings she had been operated on for prolapsus uteri; about five years ago first noticed that her abdomen was increasing in size. This proved to be due to a fibroid tumor which grew to such an extent that her abdomen measured thirty-seven inches in circumference. She had coughed for about six years, but her aspect was fairly good; she weighed 137 pounds, but was nervous and impressionable; respirations were 20; pulse, 83; temperatnre, 101.1° F.

Physical examination revealed numerous moist râles on the right side, and her sputum on microscopic examination showed the tubercle bacilli.

She was given Glyco-Heroin (Smith) in conjunction with constitutional treatment, and received local electrical treatment from the hands of a specialist. At the end of eight months her abdominal measurement was reduced to thirty-three inches, cough and expectoration had entirely disappeared, as well as the moist râles, and her temperature, pulse and respiration became normal.

Whether her cough was entirely due to the lung trouble or was partially due to the uterine difficulty I was unable to determine, but granting both factors as a cause Glyco-Heroin (Smith) cured it.

---

### THE PROPHYLAXIS AND TREATMENT OF SCARLET FEVER.

BY E. W. SAUNDERS, M.D., ST. LOUIS, MO.,

Professor of Pediatrics and Clinical Midwifery, Washington University.

The time-honored principle that scarlet fever is contagious until all desquamation ceases should receive the assent of all practitioners, and the occasional dissenting voice should produce no change in our rules for isolation. I believe such statements as those made by Millard (*Lancet*, April 5, 1902) that the scales of the skin are not contagious except as they become infected from the faucial or nasal secretion, is likely to do much harm, and will result in relaxation of prophylactic measures in many quarters which can only result in a greater spread of the disease.

Of course, it has never yet been positively shown that scarlet fever is a septicemia and that its etiologic micro-organism resides in the epidermis; nevertheless, the summation of clinical experience is that a desquamating child is dangerous to others. While it is possible that the epidermis is infected from the faucial, salivary or nasal secretions and that it does not receive its germs from the blood, the clinical fact remains that many, if not all, the scales contain the scarlet fever germ, and are, therefore, dangerous to others. The difficulty of thoroughly disinfecting the skin is well known; hence, it behooves us to regard the patient as unsafe as long as particles from the skin can contaminate the air, or be conveyed to others.

Millard made the experiment of allowing children having had scarlet fever to leave the hospital in four to six weeks, and he found only 5 out of 190 who carried the disease to others.

I fear that an extended observation would have revealed others infected. From my own experience I can give a much higher percentage after six to eight weeks' isolation, and believe Millard's figures are certainly remarkable.

But it must be always understood that any persistent lesion in the nose, throat or ear may contain the scarlatinal poison much longer, and ten to twelve weeks' isolation of severe cases is not uncommon. Hence, I would reiterate as the first principle of prophylaxis that the patient must be isolated from six to twelve weeks. This isolation must be rigid in all its details.

Toward the end of the isolation period the liberal use of antiseptics on the desquamating parts is necessary. Of these chloride of lime, dilute alcohol, solutions of corrosive sublimate or formalin are to be recommended. The application of carbol vaseline to remove the scales once or twice daily is very effective. The lesions in the nose should also be cleansed and some antiseptic ointment applied.

Having removed and disinfected all the possible sources of contagion on the patient, the next difficulty is to disinfect the patient's room and its contents. Here is where the great difficulty arises. My own experience can not give much encouragement to the enthusiastic supporters of the formic aldehyde disinfections. The disinfection of the house by fumigation with formic aldehyde gas is almost useless as far as the destruction of the scarlatinal virus is concerned. In the last few years I can recall several cases of recurrence as soon as children were allowed to re-enter the infected house. Judging from my own clinical cases, the fumigation with sulphur is more effective than formic aldehyde to destroy the scarlet fever germs.

The ordinary fumigation should not be considered sufficient. The whole room must be scoured and washed with corrosive sublimate, chloride of lime, etc. The furniture should be washed and given a prolonged airing, and the room should be closed to outsiders for a prolonged period.

The bedding and clothing should be such that they can be boiled or destroyed. It is taken for granted that all unnecessary carpets, curtains and furniture were removed when the room was selected as an isolation place.

The removal of the sick child to a room in a hospital, built expressly for contagious diseases, has its distinct advantage, in that it limits the spread of the disease; and McCollum has done good service in insisting that the number of cases can be diminished if the child is isolated in a hospital at once. But the ordinary hospital has one grave disadvantage—this is the liability to carry secondary infection from one child to the other. In wards in which ten to fifteen patients are placed, the possibility of some virulent streptococcus being carried from one child to the other is always imminent, and no amount of care in selecting the cases will entirely obviate the danger. It is on this account mainly that the mortality in the hospitals is high. McCollum, at the South Department of the Boston City Hospital, reported that the usual mortality is near 10 per cent. In private practice the mortality, judging from twenty-five years' experience, is not over 1 to 2 per cent.

These figures are sufficiently wide apart to make us hestiate to send a child to any place where another child has the same disease. A hospital for contagious diseases, especially scarlet fever, should be so arranged that each patient occupies a separate compartment, which has a separate opening for the ingress and egress of air. The nurses, furthermore, must use the most painstaking precautions not to carry the disease germs from one patient to the other.

As to active medical treatment I have nothing new to add to what I have written elsewhere. Pilocarpin is a very satisfactory remedy in my hands. Under its use the disease, as a rule, is mild and devoid of danger. For the cerebral excitement in the early stage nothing is so valuable as chloral hydrate. In the stage of general sepsis from secondary invasions, the mineral acids are very helpful, especially dilute sulphuric acid. I have found Laveran's solution (quinin hydrochlor. 3, antipyrin 2, water 6), used hypodermically, very helpful when the temperature is very high and persistent.

The serum treatment, while not so effective as the antitoxin treatment in diphtheria, is still of great value in certain cases. The recent sera prepared from the virulent streptococci taken from the blood of patients suffering from malignant scarlatina (Moser, Baginsky) should be used in all severe cases; for by this means many a life will be saved which would otherwise succumb.

In a few cases, in which I used the serum, the results were very gratifying, and demonstrated that at least some valuable complements were added to the blood.—*Courier of Medicine.*

---

### AN INTERESTING CLINICAL CASE.

X, a white woman, twenty-two years of age, was taken into the hospital on account of syphilitic skin disease (roseola papula); a blennorrhagic vaginitis of most violent description with strong congestion of the mucous membranes of the vagina. The latter was of violent hue, somewhat brittle, and yielded abundant secretion of a greenish yellow pus, which showed under bacteriological examination abundant colonies typical of gonococcus, diplococcus and other varieties of bacteria. The gonococci infection reached to the neck of the uterus whose tissues suffered from the same degeneration as the vagina. Above the mouth of the neck,—from which a greenish yellow and somewhat thick pus oozed—was a syphilitic ulcer of the size of a dime, clean at the bottom, livid in color and rather deep.

Upon careful examination, the patient was found to be pregnant in the third month; and from the start, was subjected to energetic treatment as a serious case.

Under the treatment employed she improved rather well; but, though the blennorrhagia was not cured, the syphilitic manifestations of the skin disappeared, and the ulcer at the neck improved somewhat, until confinement, which took place at the eighth month, five months after her admission.

The confinement was normal. However, the patient was attacked by a great flux and suffered a complete laceration of the right side of the neck; an incomplete laceration of the left side; an incomplete laceration of the rear wall of the vagina; and a two-thirds laceration of the perinæum. The placenta was removed at once; ample warm washes of a one per cent. solution of permanganate of potash were applied and the uterus was stimulated by massage, but remained inert. All this was reported to me by the house physician. I arrived at the hospital four hours later in company with the well-known gynecologist, Dr. Mendez Capote, who, upon having examined the patient, decided to sew up the lacerations. He washed out the vagina and uterine cavity completely; adjusted with the scissors the edges of the lacerated tissues; sewed up the wounds and touched the ulcer at the neck with the cauterizer; then he gave another wash and plugged with iodoform gauze.

When the patient was on the operating table she had fever, 38.4° C. At five p.m. the fever wast 39°; then the vaginal plug was taken out and a great intra-uterine wash of a one-half per cent. solution of permanganate was applied very hot in a quantity of five liters. The fever was at 40° throughout the night, and washes were given every four hours.

The following day, at 8 a.m., temperature 40°, same local treatment. The fever lasted all day, falling to 39° by the wash, but rose again to 40°.

The day thereafter, fever at 41°; same treatment with more vaginal washes of bichloride of mercury, before the uterine washes; the fever keeps on at 41°.

On the next day at 8 a.m. (temperature 41.5°), I took out the stitches made on the day of confinement, washed well both uterus and vagina, dried the latter with carbolated cotton and conveyed into the uterine cavity eight grammes of pure Hydrozone, taking care that this liquid should flow towards the vagina, into which I poured about 60 grammes of the same liquid and drained the uterus with simple gauze saturated in Hydrozone, while the vagina was drained by the same means.

From that time on the fever declined slowly, and at 6 p.m. it was apyretic. The fever did not return and the patient's cure proceeds without further difficulty.

This case, which is interesting by itself, proves of great value in setting forth two points; viz.:

1. that, although the intra-uterine injections of pure Hydrozone may be dangerous, it can be applied if care is taken to keep the neck dilated as much as possible.

2. That in this case the superiority of Hydrozone over the other treatments of puerperal septicæmia, in connection with gonococcia, is indisputable; and that this splendid result should encourage repetition of its application.*—Dr. Matias Duque, Director of the San Antonio Hospital, Section of Hygiene. Abstract from the *Revista Medica Cubana*, April 15, 1903.

---

### SPRING-WATER AND TYPHOID FEVER.

The firmly rooted popular belief in the superior excellence of ground-water, and especially of spring-water, is not difficult to understand. The usual clearness and brilliancy of such waters, together with their coolness, render them highly attractive to the eye and pleasing to the taste. In times of typhoid epidemics "pure spring-water" is a shibboleth of undoubted commercial value. A strong and wholly natural preference for ground-water as a source of town-supply has been evinced on many occasions. The citizens of Ithaca, after becoming fully aware of the origin of the late typhoid epidemic, could not express too deeply their abhorrence of surface-water. In France for many years *les eaux des sources* have held the highest position in the estimation of most municipal authorities, and in that country spring-waters have been

* The son of the patient suffered from blennorrhagia in the eyes. He was treated with one-fourth solution of permanganate and installations of pure Hydrozone twice daily, alternating with cauterizations of forty per cent. solution of nitrate of silver; and he kept his sight.

widely accepted as the most desirable source of municipal supply. The last decade, however, has seen a profound change in the position accorded by sanitarians to spring-waters. From many quarters evidence has accumulated that the delectable appearance of spring-water too often masks a treacherous nature. Gärtner ("Klinisches Jahrbuch," ix, 1902), in a recent monograph on this subject, has brought together a formidable array of outbreaks of typhoid fever attributed with greater or less certainty to spring-water. He clearly shows that in many instances the trusted spring-water is nothing but unfiltered surface-water of dubious antecedents. In chalk and limestone regions especially, where the existence of extensive subterranean channels and crevices renders it exceedingly difficult to determine the sources from which a spring is fed, the danger of surface contamination is ever present. The calamitous typhoid epidemic at Worthing, England, in 1893, will at once occur to the student of epidemiology as a case where the fissures in chalk were responsible for an unusually extensive outbreak of disease. In some regions small brooks or rivers which are known to receive sewerage pollution may disappear wholly or in part in openings in their beds, only to emerge at a remote point as gushing and copious "springs" which confiding inhabitants will make use of for drinking purposes with a calm assurance of safety which would indeed be ludicrous if it did not so often lead to appalling consequences. The changeable character of spring-water, both as regards turbidity and bacterial content; its dependence upon the amount of rainfall, upon the topography of the country and the underlying geological strata ; and, above all, the often insuperable difficulty of learning the real origin of the water are not features that increase its desirability as a source of supply. Gärtner points out that geologists are largely responsible for the growth of the modern views regarding the uncertain character of spring-water, and quotes with unqualified approval a statement by the geologist, Heim, that he would drink the filtered water of Lake Zurich with a feeling of greater security than the water from the majority of springs with which he was acquainted. "Springs are changeable—to-day good, to-morrow, perhaps, unsafe."

If a wide-spread reaction has already set in against the indiscriminate use of ground-waters it is amply justified. There have been several noteworthy illustrations of the unsafe character of spring-water in the hygienic annals of recent years. The water supply of Paris, which is perhaps the most extensive modern system of municipal spring-water supply, was for a time highly vaunted as an example of a naturally pure water, as compared with the "merely purified" water of Berlin and other European capitals. Pure spring-water was eloquently contrasted with filtered surface-water; innocence was said to be better than repentance. It was not long after its introduction, however, before the Paris supply fell under a cloud. The epidemics of typhoid fever in 1894 and 1899, and the searching investigations to which they gave rise, have shown unmistakably that the spring-water from the region of the Vanne is not only not beyond suspicion, but that it is actually responsible for an excessive amount of typhoid fever in Paris. The facts brought to light by these and similar inquiries have caused a decided change in the opinion of French sanitarians regarding the character of spring-water, and recent utterances by Brouardel and others indicate that in France spring-water has had its day as the ideal for a supply.

In addition to the epidemiological evidence, recent bacteriological investigation has also cast doubt on the universal wholesome character of waters of this class. Horton (*Journal of Hygiene*, vol. iii, p. 155, 1903) has reported finding the colon bacillus in water from a number of springs and driven wells, and concludes, as the result of his investigations, that the fact that water is derived from a drilled well can in no wise be taken as a guarantee of its potability.

We can no longer hesitate to admit the fact that many spring- and ground-waters are nothing but insufficiently filtered surface-waters which may, indeed, have traversed long distances underground, but which have met in their course no layer of soil or sand of purifying power. To continue to place implicit trust in waters of this sort is to tempt further "accidents" of a kind with which medical literature already teems.—*Editorial* (*Journal of the American Medical Association, July 4, 1903*).

### NEUROSIS.

Of the various pathological conditions none confronts the busy practitioner of medicine more frequently than nervous affections. They are associated with the common every-day ills of life. The complicity of the nervous system, its manifold functions and extensive disturbances, render its diseases more varied than those of other systems, and so much on the increase that our European brethren give it the name of Neurosis Americanne.

The various causes that produce it are too numerous to mention, and we have at our disposal a number of valuable remedies—the official preparations of the United States Dispensatory, the action of which is well understood, as strychnia, the preparations of zinc, phosphorus, the bromides and a host of others that have stood the test of time. Then, again, we have a number of proprietary preparations that deserve favorable mention and form quite an important part of a doctor's armamentarium.

Several months ago my attention was called by a gentleman of learning and ability, who was quite prominent in the medical profession, to a preparation called Neurilla, manufactured by the Dad Chemical Company of New York. A study of its component parts convinced me that it must be a remedy of great value. An epidemic of influenza, with nervous complications, gave me an opportunity to test its merits, with such brilliant results that I prescribed it as a routine measure with entire satisfaction to myself and patients. I was recently called to see an infant child of Mrs. T., the wife of quite a prominent attorney. She informed me that she was almost crazy from insomnia, that her babe, then two years old, seemed to have inherited neuralgic tendencies; that from birth she had been a victim of insomnia, a perfect bundles of nerves, who never slept more than a few minutes at a time; that when apparently well she was always nervous and exhibited a restless disposition, and kept her nurse moving around all the time; that she had been treated by a nnmber of specialists, all to no purpose. I felt at a loss to formulate a theory as to the pathological conditions, but I prescribed Neurilla in heroic doses and from the first night I had the pleasure of seeing a decided change in the baby's condition, so continued it in thirty-drop doses, three times daily and bedtime, and, remarkable to relate, with no other treatment the child went rapidly on to a complete recovery, gained rapidly in weight and strength, and as to sleep has become a regular Rip Van Winkle. Her general health is excellent and a return of her trouble seems hardly probable.

I prescribe Neurilla almost daily for teething children, and for the many ills during the period of dentition it is the remedy "par excellence," and I have discarded the various anodynes and nervines that once entered into our prescriptions and so often failed to have the desired effect, and for that nervous condition attending pregnancy it stands without a par. In alcoholic excesses no remedy ever employed by me has given better results. In fact, my experience with it has been so favorable in general practice that I felt it a duty to give this expression of its value to my brethren. I am now combining it with the bromides in cases of epilepsy with decided success, always increasing the interval and allaying the violence of the attack, but to enumerate the various conditions in which it is indicated would require much space. In a practice of thirty years I have had excellent opportunities to test the virtues of a great many products of our manufacturing chemists and I feel no hesitancy in saying that I regard it as the finest product of its class of modern pharmacy. A study of its formula, as well as witnessing its good effect at the bedside, is all that is necessary to endorse every claim made for it.

J. A. KNIGHT, M.D.

Eatonton, Ga.

---

### A NEW DEPARTURE.

In these days when a gullible public prescribes for itself from the patent medicines on the frieze of the trolley-cars, or takes the profitable substitution that the druggist passes over the counter, it is no wonder that physicians feel a bit out of sympathy with the vender of drugs, and make unfavorable comparisons between the commercialism of the men who supply medicines and the science of the medical profession that prescribes them.

But we should never forget that were it not for the great manufacturers and importers of drugs we might still cull our own herbs and use our own mortars and pestles. As an indication of the aid that such houses may be to physicians, we call attention to the colored plates of pathogenic organisms that have been prepared for the profession by the house of M. J. Breitenbach Co., the importers of Gude's Pepto-Mangan. By their permission we have inserted a few of the set of sixty in our advertising pages.

No text-book and no one work on pathogenic bacteria contains such a number of excellent diagnostic illustrations, nor such beautiful examples of lithographic art, as these.

Many physicians are too far from libraries and laboratories to be able to put into practice the training of their college days. They need just such a set of reference plates to be able to make microscopical examinations. The recognition of this need and the care that has been taken to fill it shows a spirit of enterprise in this firm that we wish might serve as an example to others. For, if, instead of advertising to the public, the manufacturers of drugs would make such valuable contributions to science as lies in their power, there might be more sympathy between them and physicians.

The full set of sixty cuts has been prepared to send to any physician who writes for them, from the firm of M. J. Breitenbach Co., New York.

### CROUP REMEDY.

BY N. F. HOWARD, M.D., DAHLONEGA, GA.

Compound syrup squills ................................ v ʒ

Tincture lobelia ................................ i ʒ

Wine ipecac ................................ ii ʒ

M. Sig.—Dose for children, from 8 to 60 drops. If the croup has the ring of the crow cough, the dose should be repeated every half-hour until there is nausea and emesis, or until relief is given. There should be no delay if this croup cough continues and should be added grain doses of calomel and quinine, and should be given every hour or two, and also a sear-cloth of sweet oil, camphor and turpentine should be applied to the chest. When the severity of the attack is broken, small doses may be given as an expectorant, and also simple medicines such as soda and syrup and alum and syrup may be given.

I have used this croup remedy for many years with satisfactory results.

